# Obstructive ureteric calculus with superimposed infections causing reversible posterior leukoencephalopathy syndrome: A case report

**DOI:** 10.1097/MD.0000000000025589

**Published:** 2021-04-23

**Authors:** Fei Xie, Yanli Cai, Lin Huang, Jianqiang Hao, Tianjin Ling, Seidu A. Richard

**Affiliations:** a Department of Neurosurgery; b Department of Neurology; c Department of Cardiology, The First People's Hospital of Ziyang, No. 66, Rende west road, Ziyang, 641300, Sichuan, PR China; d Department of Medicine, Princefield University, P.O. Box MA 128, Ho-Volta Region, Ghana, West Africa.

**Keywords:** calculus, encephalopathy, hypertension, leukoencephalopathy, pneumonia, tinnitus

## Abstract

**Rationale:**

Reversible posterior leukoencephalopathy syndrome (RPLS) is a clinicoradiological phenomenon first observed 2 decades ago. Reversibility is the hallmark of this rare clinical phenomenon once the triggering pathology is aptly and adequately treated. Tinnitus preceding bilateral hearing loss as a symptomatology of RPLS has not been reported in the literature. Furthermore, chronic obstructive ureteric calculus with superimposed infections as a cause of RPLS has not been reported in the literature.

**Patient concerns:**

A 57-year-old female was admitted at our facility because of 2 days history of hearing loss in both ears. She experienced tinnitus in both ears 2 weeks prior to the hearing loss. She is a known hypertensive. She has also undergone multiple surgical treatments for urinary calculi.

**Diagnosis:**

Computed tomography (CT) scan of the urinary system revealed a calculus at the right ureter. Magnetic resonance imaging (MRI) showed abnormal signals at both temporo-parieto-occipital (TPO) cortices, the subcortical area, as well as the left hippocampus which was consistent with the diagnosis of RPLS.

**Interventions:**

While on antibiotics for treatment of infections, the patient went into hypertensive encephalopathy and pneumonia was also established necessitating intensive care.

**Outcomes:**

We observed a resolution of the patient's temperature and hypertension when the right ureteric stone finally descended into the bladder. Also, we observed disappearance of the abnormal signals at both TPO cortices, the subcortical area, as well as the left hippocampus. Two years follow-up revealed no recurrence of her symptomatology.

**Lesions:**

Patients who present with hypertensive encephalopathy maybe more prone to developing RPLS. Renal insufficiency alone or hypertension alone may not be single predisposing entities to RPLS but rather multiple predisposing factors.

## 1 Introduction

Reversible posterior leukoencephalopathy syndrome (RPLS) is a clinicoradiological phenomenon first observed 2 decades ago.^[1,2]^ This disorder is mostly observed in patients with renal insufficiency, hypertension, immunosuppression (cytotoxic drugs), autoimmune disorders, and pre-eclampsia or eclampsia.^[2–4]^ Pathologically, the syndrome is characterized by reversible subcortical vasogenic brain edema.^[5,6]^ Clinically, the patients often present with acute or subacute neurological symptoms such as seizures, encephalopathy, headache, visual disturbances, as well as altered mental status with one or more of the underlying medical conditions above.^[3,4,7]^

Classically, neuroimaging studies usually reveals vasogenic edema involving the white matter in the posterior regions of the cerebral hemispheres, principally the bilateral parieto-occipital regions.^[2,5,6,8,9]^ Computed tomography (CT) scan, magnetic resonance imaging (MRI), and diffusion weighted images are usually the most preferred radiological modalities used to detect this phenomenon.^[6,10,11]^ Reversibility is the hallmark of this rare clinical phenomenon once the triggering pathology is aptly and adequately treated.^[6,7]^

We present a rare occurrence of RPLS in a known hypertensive patient with an obstructive ureteric calculus superimposed with infections. Tinnitus preceding bilateral hearing loss as a symptomatology of RPLS has not been reported in the literature. Furthermore, obstructive ureteric calculus with superimposed infections as a cause of RPLS has not been reported in the literature.

## 2 Case report

A 57-year-old female was admitted at our facility because of 2 days history of hearing loss in both ears. She experienced tinnitus in both ears 2 weeks prior to the hearing loss. She has undergone multiple surgical treatments for urinary calculi. She is also a known hypertensive for the past 5 years. Electrocardiogram (ECG) also detected coronary heart disease 4-years after establishing the hypertension. She was admitted at a local hospital a month prior to the above symptomatology due to severe urinary tract infection (UTI) with thrombocytopenia. She was treated with intravenous (IV) antibiotics for 5 days and discharged home. General cranial nerve examination did not yield much. However, Webber and Renner tests revealed severe left side hearing loss and a mixed hearing loss in the right ear. General physical examination was unremarkable. Routine chest X-ray was normal. Also, routine laboratory investigation at the current presentation was grossly normal.

**Figure 1:**
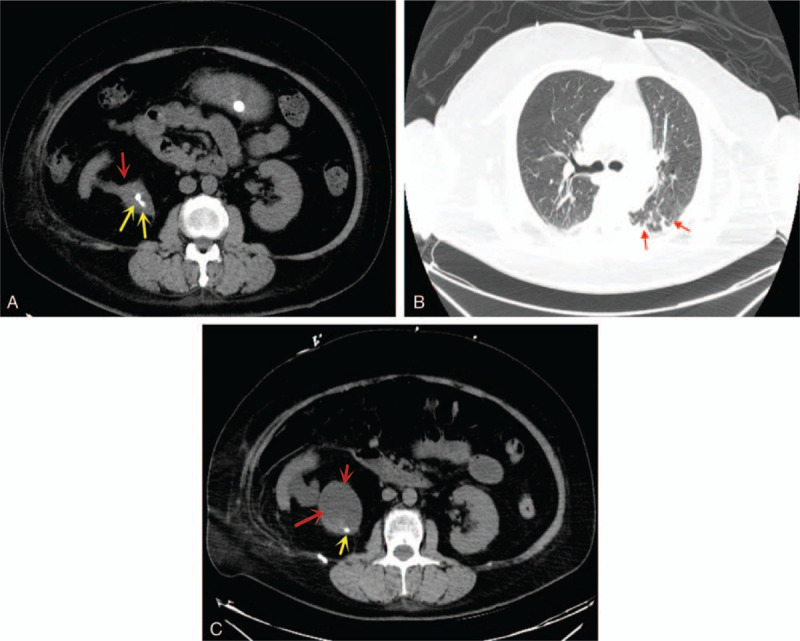
(A) Is an CT scan of the urinary system showing a calculus at the right ureter. Red arrow = ureter, yellow arrows = stones. (B) Is an emergency chest CT showing left lung pneumonia. Red arrows = pneumonia. (C) Is a repeated CT scan of urinary system showing a descend of the right ureter stone with hydronephrosis. Red arrow = ureter, yellow arrows = stone.

CT scan of the urinary system revealed a calculus at the right ureter (Fig. 1A). The stone was located at 2nd and 3rd lumbar vertebral body planes. Also, the whole right ureter was slightly dilated. She was given hyperbaric oxygen and intramuscular dexamethasone at a maximum daily dose of 16 mg for 4 days and reduced to a maximum daily dose of 8 mg for another 3 days in attempts to resolve the sudden deafness. She also complained of backache, chills, and fever. Thus, a diagnosis of UTI secondary to ureteric calculi was made and she was put on IV Ceftriaxone Sodium 2 g daily for 5 days.

**Figure 2:**
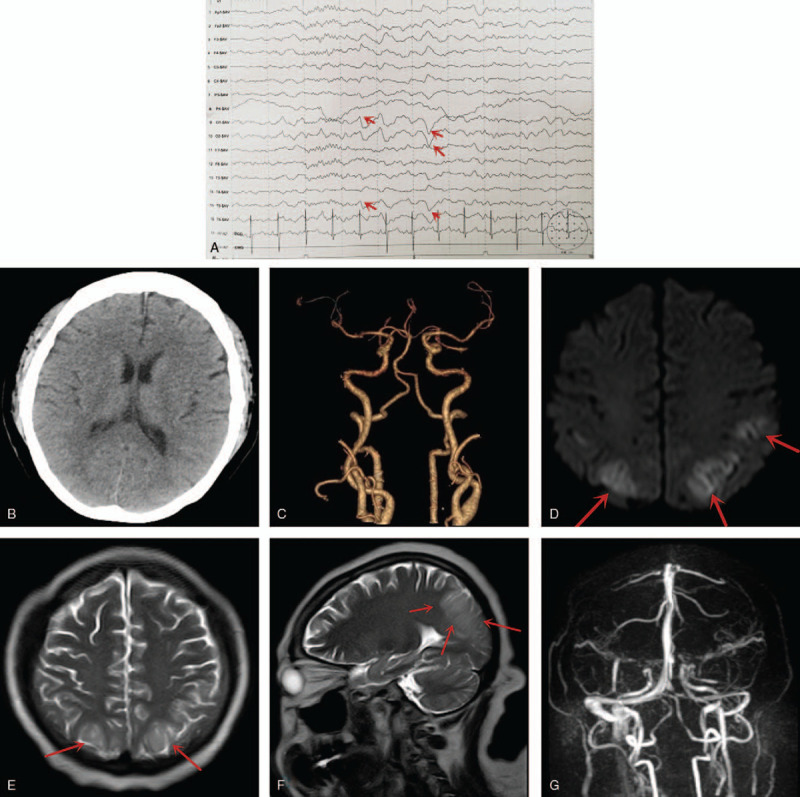
(A) Is an EEG showing abnormal occipital lobe apical waves consistent with acute seizure disorder. Red arrows = abnormal waves. (B and C) Are head CT (B) scan and CTA (C) showing no abnormalities. (D–F) Are MRIs showing abnormal signals at both temporo-parieto-occipital cortices, the subcortical area, as well as the left hippocampus. (G) Is an MRA showing no vascular abnormality. CT = computer tomography, CTA = CT angiography, MRA = magnetic resonance angiography.

Her condition suddenly aggravated 5 days after establishing the UTI although she was on antibiotics. The patient went into hypertensive encephalopathy. She was transferred to the intensive care unit (ICU) immediately and an emergency chest CT showed left lung pneumonia (Fig. 1B), while a repeated CT scan of urinary system showed a descend of the right ureter stone with hydronephrosis (Fig. 1C). Blood as well as urine cultures and sensitivities revealed staphylococcus hemolyticus which was sensitive to piperacillin, tazobactam, and levofloxacin. Therefore, she was put on the above antibiotic to treat both UTI and chest infection. Two days later, she developed seizure so we performed EEG at the bedside which revealed abnormal occipital lobe apical waves consistent with acute seizure disorder (Fig. 2A).

Head CT scan and CT angiography (CTA) showed no abnormalities (Fig. 2B and C) while MRI showed abnormal signals at both temporo-parieto-occipital (TPO) cortices, the subcortical area, as well as the left hippocampus (Fig. 2D–F) which was consistent with the diagnosis of posterior leukoencephalopathy syndrome. Magnetic resonance angiography (MRA) also showed no vascular abnormality (Fig. 2G). Also, cerebrospinal fluid examinations were grossly at normal ranges. She was put on dexamethasone injection 16 mg 6 hourly for 3 days. The posterior leukoencephalopathy syndrome improved remarkably.

**Figure 3:**
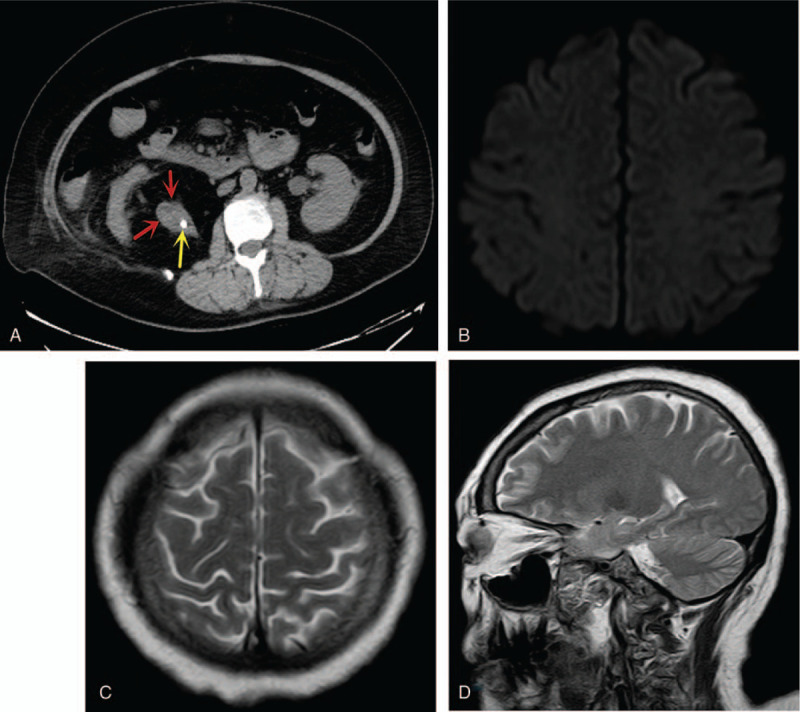
(A) Is a CT scan showing a total descend of the right ureteric stone into the bladder and a resolution of the dilatation of right ureter. Red arrow = ureter, yellow arrows = stone. (B–D) Are repeated MRIs of the head showing disappearance of the abnormal signals at both TPO cortices, the subcortical area, as well as the left hippocampus. CT = computer tomography, TPO = temporo-parieto-occipital.

Five days later, pelvic CT scan revealed a total descend of the right ureteric stone into the bladder and a resolution of the dilatation of right ureter (Fig. 3A). Also, the patient's temperature returned to normal range. Furthermore, a repeated MRI of the head revealed disappearance of the abnormal signals at both TPO cortices, the subcortical area, as well as the left hippocampus (Fig. 3B–D). Thus, a definitive diagnosis of RPLS secondary to obstructed right ureteric calculus with superimposed UTI and left lung pneumonia was made.

The patient's hearing loss was also restored and she was discharged home after 2 months stay in admission in the hospital. Her pre-existing hypertension which was also high at time of admission was adequately controlled. Two years follow-up revealed no recurrence of her symptomatology. Nevertheless, she is on follow-up because of possible recurrence of the renal stones which could also cause a recurrence of the leukoencephalopathy syndrome.

## 3 Discussion

RPLS was first observed in 1996 in patients who had renal insufficiency, hypertension, or were immunosuppressed.^[1,2]^ Current studies have shown that, all age groups are susceptible to the syndrome.^[6,10]^ Also, studies have shown that, females are more prone to developing this phenomenon than males even when eclampsia cases were excluded.^[3,10,12]^ Bartynski and Boardman observed renal failure as the common underlying medical condition found in patients diagnosed with RPLS.^[13]^ Furthermore, nephrotic and or nephritic conditions often associated with hypertensive, severe hypoproteinemia, as well as massive edema with fluid retention coupled with increased vascular permeability is the key predisposing factor for RPLS.^[2,5,6]^ Our patient had undergone multiple surgical treatments for urinary calculi and her index presentation was as a result of another calculi.

Hypertension was almost always observed in patients who developed RPLS.^[5,6,10]^ Patients who present with hypertensive encephalopathy maybe more prone to developing RPLS.^[5,6,8,14]^ Our case demonstrates that, hypertension may not be a single predisposing entity to RPLS but rather multiple predisposing factors with hypertension inclusive. Studies have shown that, in 70% of patients, the onset of RPLS was preceded by acute hypertension as well as infections.^[15,16]^ Our patient was a known hypertensive patient with obstructive ureteric calculus superimposed with infections. Her hypertension was adequately controlled although it was high at time of admission. Anatomically, the bilateral parietal and occipital lobes are the key brain regions mostly affected although, the frontal lobe, temporal lobe, cerebellum, as well as brainstem regions have also been observed.^[5,6,9,14,17]^ Clinically, the patients often present with acute or subacute neurological symptoms such as seizures, encephalopathy, headache, visual disturbances, as well as altered mental status.^[5,6,14]^ The single most cardinal symptomatology in our case was tinnitus preceded by bilateral hearing loss.

Although the precise pathophysiology of RPLS is still a matter of debate, the radiological findings are often associated with distorted cerebrovascular autoregulation, endothelial dysfunction, as well as vasospasm linked to acute increases in blood pressure (BP).^[5,6,14]^ It was observed that, precipitous raise in systemic BP in patients with hypertension causes cerebrovascular autoregulation failure as well as breakdown of the blood brain barrier, leading to hyperperfusion.^[5,6]^ The parieto-occipital regions are mostly affected during the loss of autoregulation because there is lessened sympathetic innervation in the posterior cerebral arterial circulation.^[5,6,8]^ Furthermore, the loss of cerebral autoregulation often results in arteriolar vasodilation as well as endothelial dysfunction, leading to transudation of fluids and proteins which intern triggers cerebral vasogenic edema.^[5,6,8]^ Also, cerebral vasospasm was capable of causing hypoxia and cytotoxic ischemia resulting in edema at the parietal-occipital regions as well as watershed regions of the brain ^[5,6,8,14]^

Apart from hypertension and renal diseases, other conditions that have implicated as predisposing factors to RPLS includes, immunosuppressive drugs, fluid overload, autoimmune diseases, pre-eclampsia/eclampsia, and sepsis.^[3,4]^ Cases of human immunodeficiency virus (HIV) patients presenting with PRLS has also been reported.^[7,18]^ It is wealth noting that, HIV maybe an independent risk factor for endothelial dysfunction as well as vascular disease.^[7,18,19]^ Azotemia may trigger interstitial brain edema via augmentation of capillary permeability as well as cytotoxic edema resulting in direct injury to the brain parenchyma in patients with renal failure.^[20]^ RPLS has also been reported in dialysis-dependent patients as a result of uremia and not dialysis disequilibrium syndrome that often occurs few hours after treatment.^[20,21]^ Sunitinib, a tyrosine kinase inhibitor has been implicated as a cause of RPLS.^[22]^

Radiologically, brain imaging typically shows vasogenic edema in the parieto-occipital regions of both cerebral hemispheres.^[5,6,8,11]^ Also, the subcortical white matter and the cortices are often involved and the associated edema is mostly asymmetric, but almost always bilateral.^[5,6,8]^ CT scans often reveal vasogenic edema with a bihemispheric distribution.^[6,17]^ On the other hand, MRI often shows hyperintense signaling on T2-weighted, hypointense signaling in T1-weighted images, as well as fluid-attenuated inversion recovery images consistent with cerebral edema.^[6,7,13]^ The consistent vasogenic edema on MRI often follows a parieto-occipital pattern.^[13]^ Although frontal sulcus or watershed pattern has also been observed, 70% of all patients often present with parieto-occipital distribution.^[6,13]^ Furthermore, although less frequent, RPLS has been observed in areas such as the cerebellum, brain stem, basal ganglia, and the spinal cord.^[6,9]^

In our case, MRI showed abnormal signals at both TPO cortices, the subcortical area, as well as the left hippocampus. Studies have also demonstrated blood vessel irregularities consistent with vasoconstriction using CTA, MRA, and catheter cerebral angiogram in patients with RPLS. In our case, CTA and MRA did not show any vascular anomalies. There are no precise treatments for RPLS.^[5,6,8]^ The lesions frequently reverse when the triggering factors are eliminated or treated.^[5,6,8]^ Seizures are managed with antiepileptic medications.^[5,6,8]^ In our patients, the treatment was specific for the underlying condition like infection, hypertension, as well as the watchful waiting of a relief of the obstructed right ureteric stone. We observed a resolution of the patient's temperature and hypertension when the right ureteric stone finally descended into the bladder. Also, we observed disappearance of the abnormal signals at both TPO cortices, the subcortical area, as well as the left hippocampus. Intraparenchymal hemorrhage and sulcal subarachnoid hemorrhage are the most common complication-associated RPLS.^[6,23]^ Studies have shown that approximately 18% to 30% of patients with RPLS often develop both types of hemorrhages.^[6,23]^ We did no observe any hemorrhagic complication is our case.

## 4 Conclusion

The occurrence of RPLS in a known hypertensive patient with obstructive ureteric calculus superimposed with infections is very rare. Patients who present with hypertensive encephalopathy maybe more prone to developing RPLS. Tinnitus preceding bilateral hearing loss could be a warning sign of RPLS. Renal insufficiency alone or hypertension alone may not be single predisposing entities to RPLS but rather multiple predisposing factors.

## Author contributions

**Conceptualization:** Fei Xie, Yanli Cai, Lin Huang, Jianqiang Hao, Tianjin Ling, Seidu A. Richard.

**Data curation:** Fei Xie, Yanli Cai, Lin Huang, Jianqiang Hao, Tianjin Ling, Seidu A. Richard.

**Formal analysis:** Fei Xie, Yanli Cai, Lin Huang, Jianqiang Hao, Tianjin Ling, Seidu A. Richard.

**Investigation:** Fei Xie, Yanli Cai, Tianjin Ling.

**Methodology:** Fei Xie, Yanli Cai, Lin Huang, Jianqiang Hao, Tianjin Ling, Seidu A. Richard.

**Resources:** Fei Xie, Yanli Cai, Lin Huang, Jianqiang Hao, Tianjin Ling.

**Supervision:** Fei Xie, Yanli Cai, Lin Huang, Jianqiang Hao, Tianjin Ling.

**Writing – original draft:** Seidu A. Richard.

**Writing – review & editing:** Fei Xie, Yanli Cai, Lin Huang, Jianqiang Hao, Tianjin Ling, Seidu A. Richard.
